# The *Shigella* T3SS needle transmits a signal for MxiC release, which controls secretion of effectors

**DOI:** 10.1111/j.1365-2958.2010.07413.x

**Published:** 2010-10-11

**Authors:** Isabel Martinez-Argudo, Ariel J Blocker

**Affiliations:** Schools of Cellular and Molecular Medicine and Biochemistry, University of BristolBristol BS8 1TD, UK

## Abstract

Type III secretion systems (T3SSs) are key determinants of virulence in many Gram-negative bacteria, including animal and plant pathogens. They inject ‘effector’ proteins through a ‘needle’ protruding from the bacterial surface directly into eukaryotic cells after assembly of a ‘translocator’ pore in the host plasma membrane. Secretion is a tightly regulated process, which is blocked until physical contact with a host cell takes place. Host cell sensing occurs through a distal needle ‘tip complex’ and translocators are secreted before effectors. MxiC, a *Shigella* T3SS substrate, prevents premature effector secretion. Here, we examine how the different parts of T3SSs work together to allow orderly secretion. We show that T3SS assembly and needle tip composition are not altered in an *mxiC* mutant. We find that MxiC not only represses effector secretion but that it is also required for translocator release. We provide genetic evidence that MxiC acts downstream of the tip complex and then the needle during secretion activation. Finally, we show that the needle controls MxiC release. Therefore, for the first time, our data allow us to propose a model of secretion activation that goes from the tip complex to cytoplasmic MxiC via the needle.

## Introduction

Type III secretion systems (T3SSs) are key determinants of virulence in many Gram-negative bacteria, including animal and plant pathogens. They are protein transport devices used to inject ‘effector’ proteins into the plasma membrane or cytoplasm of host cells to manipulate cell processes. Effectors can modulate intracellular trafficking, induce apoptosis, provoke reorganization of the cytoskeleton and manipulate the immune response (Cornelis, 2006; Galan and Wolf-Watz, 2006). In general, T3SSs or ‘secretons’ consist of four parts: a cytoplasmic ‘bulb’ or C-ring, a transmembrane region, a hollow ‘needle’ protruding from the bacterial surface, which serves as a secretion conduit, and a distal needle tip complex. The tip, needle and transmembrane region, when purified together, are known as the ‘needle complex’ or NC (Kubori *et al*., 1998; Blocker *et al*., 2001; Veenendaal *et al*., 2007).

*Shigella flexneri*, the causative agent of human bacillary dysentery, uses a T3SS to inject effector proteins that will allow invasion and dissemination through the colonic epithelium. In order to inject effector proteins into host cells, three proteins known as the ‘translocators’ (IpaB, IpaC and IpaD in *Shigella*), are required. IpaB and IpaC will assemble a pore in the host cell membrane, through which other virulence effector proteins may then be translocated (Blocker *et al*., 1999; Veenendaal *et al*., 2007). Once host cell invasion has occurred, it is followed by dissemination within the epithelium, which leads to destruction of the mucosal lining and self-limiting dysentery (Parsot, 1994; Schroeder and Hilbi, 2008).

The determinants of *Shigella* virulence are mostly encoded on a large virulence plasmid. Located within the ‘entry region’ are the *spa*, *mxi* and *ipa* operons coding for components of the type III secretion machinery, translocators and ‘early effector’ proteins, the expression of which is not controlled by T3SS activity (Parsot, 1994; Le Gall *et al*., 2005). They are expressed when the environmental conditions are appropriate for invasion (Le Gall *et al*., 2005; Schroeder and Hilbi, 2008). At this point functional type III secretion devices are built following a sequential process well-conserved between T3SSs (recently reviewed in Deane *et al*., 2009). However, secretion from the T3SS is blocked until contact with a host cell membrane generates an activation signal. Additionally, expression of ‘late effector’ genes (like *ipaH*), encoding proteins involved in later stages of infection, is controlled by the T3SS activity. Indeed, it is prevented by OspD1, an early secreted effector that represses the transcriptional activator MxiE (Parsot *et al*., 2005). MxiE transcriptional activation also requires a co-activator, IpgC, the intrabacterial chaperone of IpaB and IpaC (Mavris *et al*., 2002). Once secretion is activated, translocator proteins that will form a pore in the host cell membrane are secreted first, followed by the early effectors. Thus, IpgC is released from its substrates, OspD1 is secreted and the IpgC–MxiE complex activates transcription of the late effector genes (Parsot *et al*., 2005). Expression of a small subset of genes (including *virA*) is detected when the T3SS is not active but increases after secretion is activated (Le Gall *et al*., 2005), they will be here referred to as ‘middle effector’ genes.

A key outstanding question is how the T3SS, needle and tip complex/translocation pore work together in order to allow orderly effector translocation. In *Shigella*, it has been suggested that the host cell is sensed by the tip complex, hypothetically composed of four molecules of IpaD and one molecule of IpaB (Espina *et al*., 2006; Olive *et al*., 2007; Veenendaal *et al*., 2007; Blocker *et al*., 2008). Host cell contact would lead to membrane-insertion of IpaB, which could trigger a conformational change in the tip leading to activation of secretion (Veenendaal *et al*., 2007; Blocker *et al*., 2008). Secretion and subsequent tip recruitment of the second component of the translocon, IpaC, would allow assembly of a pore in the plasma membrane followed by secretion and translocation of effectors (Veenendaal *et al*., 2007; Epler *et al*., 2009; Roehrich *et al*., 2010). However, it is still unknown how secretion is prevented until contact with a host cell membrane generates an activation signal and how this signal is transduced to the cytoplasmic side of the T3SS to trigger its activity.

Several models that could complement each other have been proposed. The ‘extracellular plug’ model proposes that the needle tip proteins adopt a conformation that physically blocks secretion and that after host cell contact a conformational change ‘opens’ the secretion channel (Menard *et al*., 1994; Veenendaal *et al*., 2007; Blocker *et al*., 2008). The ‘intracellular plug’ model suggests that a gate-keeper family of proteins blocks the T3SS from the inside, while being partially inserted into the secretion channel. The activation signal would provoke a conformational change in the needle tip promoting export of the gate-keeper protein (Ferracci *et al*., 2005; Blocker *et al*., 2008). The ‘allosteric’ model proposes that the needle plays an active role in transmitting the activation signal from the tip to the base of the T3SS (Blocker *et al*., 2001; Cordes *et al*., 2005). This model is supported by the finding that some needle mutants show an altered tip complex and secretion phenotype (Kenjale *et al*., 2005; Torruellas *et al*., 2005; Veenendaal *et al*., 2007). However, these are all ‘one-step secretion’ models and none of them explains how differential secretion of translocators and effectors occurs, as described at least in *Shigella*, *EPEC*, *Pseudomonas* and *Salmonella* (Kubori and Galan, 2002; Kenjale *et al*., 2005; Cisz *et al*., 2008; Wang *et al*., 2008; Botteaux *et al*., 2009).

MxiC from *Shigella* belongs to the putative T3SS gate-keeper family including *Yersinia* YopN/TyeA, EPEC SepL and *Salmonella* InvE and SsaL (Pallen *et al*., 2005). In addition to sequence similarities between the proteins of this family, comparison of the MxiC crystal structure with that of YopN/TyeA from *Yersinia* confirms the high conservation of domain topology between these homologues (Deane *et al*., 2008), strongly suggesting a conserved function. Indeed, all these proteins are involved in the negative control of T3SS activity (Forsberg *et al*., 1991; Iriarte *et al*., 1998; Kubori and Galan, 2002; Day *et al*., 2003; Deng *et al*., 2004; Ferracci *et al*., 2005). Recently, Botteaux *et al*. (2009) showed that MxiC is a T3SS substrate and that an *mxiC* mutant secretes effectors constitutively in the absence of any activation signal.

We have further characterized an *mxiC* mutant and found that it additionally shows weak and delayed induction of translocator protein secretion when compared with the wild-type strain. Furthermore, we have ordered the involvement of the tip, needle and MxiC, analysing the secretion phenotype of double mutants combining specific *mxiC*, *mxiH* (encoding the needle subunit), *ipaB* and *ipaD* mutations. We also show that when compared with wild-type, the *mxiC* mutant has a similar needle tip composition. Taken together, our data lead to a novel working model of secretion activation that, for the first time, goes from the tip complex to cytoplasmic MxiC via the needle. Beyond providing important and generalizable information on regulation of T3SS activation, this work begins to establish a new paradigm for how the functional state of macromolecular machines is internally sensed and regulated.

## Results

The *Shigella* T3SS displays three different functional states (Veenendaal *et al*., 2007). ‘Leakage’ is a slow, low-level Ipa protein secretion prior to host cell contact where around 5% of Ipa proteins are secreted (Magdalena *et al*., 2002; Parsot *et al*., 2005). ‘Induction’ describes the burst of Ipa protein secretion upon host cell sensing (Menard *et al*., 1994) or addition of Congo red (CR), a small amphipatic dye molecule which acts as an artificial inducer of T3SS secretion in *Shigella* (Bahrani *et al*., 1997). This event occurs within minutes and at least 50% Ipa proteins are secreted in 15 min following CR induction (Bahrani *et al*., 1997; Magdalena *et al*., 2002; Parsot *et al*., 2005). ‘Constitutive secretion’ of Ipa proteins represents an unregulated and higher level secretion than leakage and involves not only the Ipa proteins but also the ‘late effectors’ that are involved in later stages of infection. It occurs in some needle mutants, where it is detectable only after hours (Kenjale *et al*., 2005). In the *ipaB* and *ipaD* mutant strains, constitutive secretion is much faster and detectable in minutes. It is therefore named ‘fast constitutive secretion’ (Veenendaal *et al*., 2007).

### MxiC plays a role in the induction of secretion of translocator proteins

**Fig. 1:**
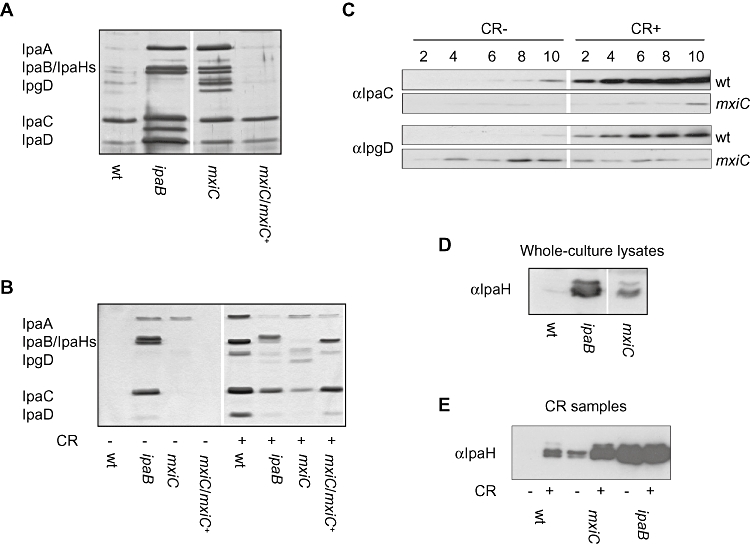
The *mxiC* mutant is impaired in induction of translocator secretion. A. Overnight leakage of *Shigella* wild-type (wt), mutant (*ipaB*, *mxiC*) and complemented (*mxiC/mxiC*^+^) strains. Supernatants were collected from overnight cultures, normalized according to cell density, separated by SDS-PAGE and silver-stained. B. Protein secretion in response to Congo red induction (CR). Cultures were grown to exponential phase, normalized by optical density and supernatants were collected from duplicates incubated 15 min at 37°C with or without the artificial inducer CR, separated by SDS-PAGE and silver-stained. Where known, the position of the Ipa proteins is indicated on the left. C. Samples from wild-type and the *mxiC* mutant were collected at the indicated times (min) after the addition of CR or not and Western blotted with the indicated antibodies. D. Total protein expression levels. Bacteria were grown to exponential phase and suspension samples were taken in order to represent the total protein fraction. Samples were normalized, separated by SDS-PAGE and Western blotted with an antibody against late effector protein IpaH. E. Proteins secreted by different strains in response to CR addition. Samples were collected as in Fig. 1B and Western blotted with an antibody against IpaH. White lines indicate where lanes were removed, all samples in the same panel were run in the same gel.

**Fig. 2:**
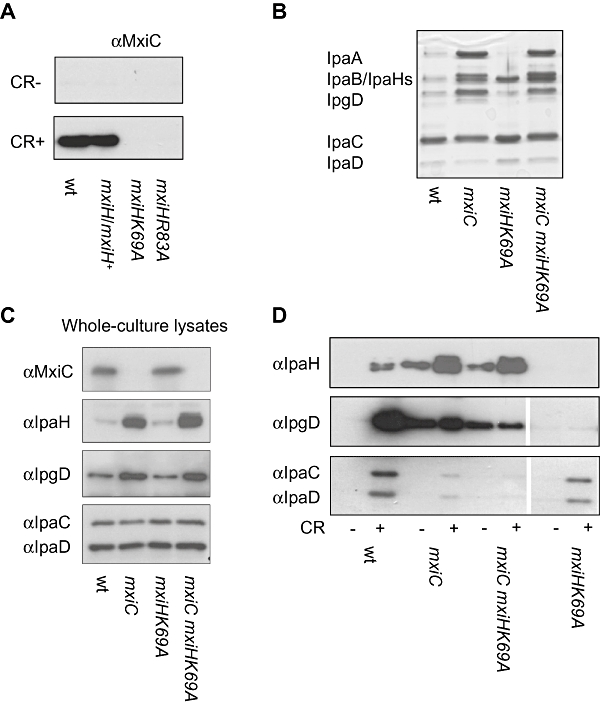
The defect in effector secretion of the *mxiHK69A* strain is due to its inability to secrete MxiC upon activation. A. Secretion of MxiC by *Shigella* wild-type and *mxiH* mutant strain complemented with several *mxiH* mutants in response to CR addition. Samples were collected as in Fig. 1B and Western blotted with αMxiC. B. Overnight leakage of *Shigella* strains. Supernatants were collected from overnight cultures, normalized, separated by SDS-PAGE and silver-stained. C. Total protein expression levels. Bacteria were grown to exponential phase and suspension samples were taken in order to represent the total protein fraction. Samples were normalized, separated by SDS-PAGE and Western blotted with the indicated antibodies. D. Proteins secreted by different strains in response to CR addition. Samples were collected as in Fig. 1B and Western blotted with antibodies against early effector IpgD, late effector IpaH and translocator (IpaC and IpaD) proteins. White lines indicate where lines were removed, all samples in the same panel were run in the same gel.

To examine the role of MxiC in secretion regulation we generated a non-polar *mxiC* deletion mutant and analysed its secretion profile. As recently reported (Botteaux *et al*., 2009), secretion of effector proteins during overnight growth (leakage) was increased in the mutant compared with the wild-type or complemented strains (Fig. 1A). To further analyse the phenotype of the *mxiC* mutant, we investigated its secretion profile following induction with Congo red. We found that the *mxiC* mutant showed much weaker secretion activation overall and especially of translocator proteins (IpaB, IpaC and IpaD) when compared with the wild-type strain (Fig. 1B). Additionally, we observed that sometimes the complemented strain did not show a fully wild-type secretion profile (Fig. 1B). Since MxiC prevents effector secretion, we reasoned that an excess of MxiC could prevent full induction of effector secretion. In fact, expression of *mxiC* from plasmid pUC19 is higher than in the wild-type strain (Fig. S1). We therefore cloned *mxiC* into the IPTG inducible plasmid pACT3 (Dykxhoorn *et al*., 1996) and titrated the IPTG concentration in order to modulate *mxiC* expression. As we expected, when MxiC expression increased we observed both a decrease in effector secretion and an increase in translocator secretion (Fig. S1). A time-course experiment showed that after CR induction the *mxiC* mutant shows only weak and significantly delayed induction of secretion of the IpaC translocator protein (Fig. 1C), while secretion of early effectors (IpgD, Fig. 1C and IpaA not shown) was constitutive. Analysis of whole extracts showed that intracellular levels of translocator and early effector genes were essentially unaffected by *mxiC* deletion (Fig. S1 and Fig. 2C; except the slight increase seen in IpgD production), as shown previously (Botteaux *et al*., 2009). As also previously described (Botteaux *et al*., 2009), the late effector protein IpaH was expressed by the *mxiC* mutant under non-activated conditions (Fig. 1D). In addition, we noticed that in the *mxiC* mutant, IpaH secretion increased after CR addition (Fig. 1E). Overall, these results suggest that MxiC positively regulates translocator release upon secretion activation, while it inhibits early release of effectors.

### The needle transmits the signal for MxiC release

The needle and its tip are involved in the control of T3SS activity as some mutants in the needle subunit (MxiH) show an altered tip complex and secretion phenotype (Kenjale *et al*., 2005; Veenendaal *et al*., 2007). Particularly interesting for us were mutants *mxiH/mxiHK69A* and *mxiH*/*mxiHR83A* (hereafter denoted as *mxiHK69A* and *mxiHR83A*), termed ‘effector mutants’ since they secrete translocators after CR addition, yet are concomitantly unable to secrete effector proteins (Kenjale *et al*., 2005), although they express wild-type levels of those proteins (Fig. 2C). MxiC prevents secretion of effectors and is secreted following induction with CR (Botteaux *et al*., 2009; and Fig. 2A). To investigate if the defect in these ‘effector mutants’ is due to an altered signal transmission between the needle and the cytoplasm that involves MxiC, we analysed MxiC secretion in strains *mxiHK69A* and *mxiHR83A*. Both strains are unable to secrete MxiC after CR addition (Fig. 2A), although they express MxiC at wild-type levels (Fig. 2C and not shown). This result identifies the regulator MxiC as an ‘early effector’-type secretion substrate that is only released after translocator secretion.

We hence hypothesized that deletion of *mxiC* in the *mxiHK69A* or *mxiHR83A* mutant background might liberate the blockage in effector secretion seen in these mutants. Furthermore, as *mxiHK69A* and *mxiHR83A* are inducible for translocator secretion, if MxiC plays a positive role in the induction of translocator secretion, an *mxiC mxiHK69A* (or *mxiC mxiHR83A*) mutant should show as weak an induction of translocators secretion as the single *mxiC* mutant. We constructed an *mxiC mxiH* mutant, complemented it with *mxiHK69A* or *mxiHR83A* and analysed the secretion profile of these strains. As expected, the *mxiC mxiH* double mutant did not secrete any of the translocator or effector proteins (data not shown). We found that *mxiC mxiHK69A* (or *mxiC mxiHR83A*, not shown) indeed secretes early effectors constitutively (Fig. 2B and D, middle panel). In addition, secretion of the late effector IpaH showed the same pattern as in the *mxiC* mutant itself (Fig. 2D, upper panel). On the other hand, as we expected, the *mxiC mxiHK69A* (or *mxiC mxiHR83A*, not shown) showed as weak an induction of translocators secretion as the single *mxiC* mutant (Fig. 2D, bottom panel). These data therefore also point to an important role for MxiC in translocator release. In addition, they directly implicate the needle in the release of MxiC, once the translocator proteins have been secreted, which in turn allows effector secretion.

### MxiC acts downstream of the needle tip in the regulatory cascade leading to secretion activation

**Fig. 3:**
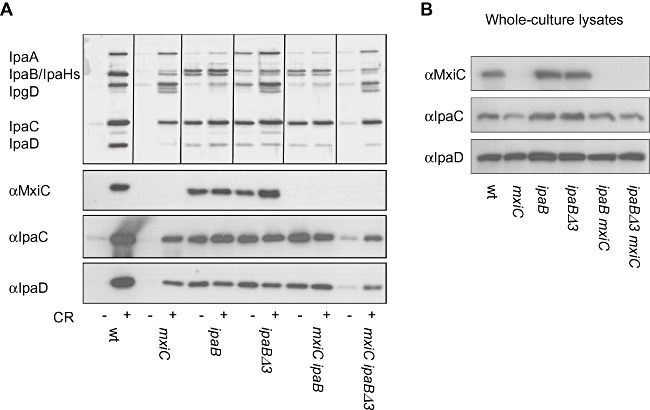
MxiC's function is dominant in the *ipaBΔ3* mutant. A. Analysis of proteins secreted by *Shigella* wild-type, mutant (*mxiC*, *ipaB* and *ipaBΔ3*) and double mutant (*ipaB mxiC* and the same complemented with *ipaBΔ*3) strains after CR induction. Samples were collected as in Fig. 1B, silver-stained (top panel) and Western- blotted with antibodies against MxiC and translocator proteins IpaC and IpaD (bottom panels). Lines are only used as visual aids. B. Total protein expression levels. Samples were collected as in Fig. 2C and Western blotted with the indicated antibodies.

In order to determine the position of MxiC in the regulatory cascade leading to secretion activation, we wanted to establish whether the function of IpaB, a tip complex component, is dominant over that of MxiC. For this, we used a previously generated *ipaB* mutant carrying a C-terminal three-amino-acid deletion (*ipaBΔ3*) that behaves as a constitutive secretor, yet remains invasive and recruits IpaC to the needle tip (Fig. 3A; Roehrich *et al*., 2010). We found that the *ipaBΔ3* mutant secretes MxiC in non-activated conditions (Fig. 3A) and we hence hypothesized that it is locked in a secretion ‘on’ conformation (i.e. similar to the secretion activated wild-type). We deleted *mxiC* in this background and analysed its secretion phenotype following CR induction in order to assess MxiC's role in translocator protein secretion. Unlike *ipaBΔ*3, that secretes the IpaC and IpaD translocator proteins constitutively, the double mutant *mxiC ipaBΔ3* showed as weak an induction of translocator secretion as *mxiC* (Fig. 3A, anti-IpaC and IpaD Western blots), though both strains express similar levels of translocator proteins (Fig. 3B). Overall, these results suggest that MxiC's function is dominant in the *ipaBΔ3* mutant and required for translocator secretion.

*ipaB* and *ipaD* mutants constitutively secrete effectors and translocators (Menard *et al*., 1994). We therefore wondered whether MxiC still plays a role in translocator secretion in these mutants that do not have a complete tip complex (Veenendaal *et al*., 2007). For this, we made *mxiC ipaB* and *mxiC ipaD* double mutants and analysed their secretion phenotype following CR induction. The double mutants have the same phenotype as the single *ipaB* or *ipaD*: constitutive secretion of translocators and effectors and unresponsiveness to CR (Fig. 3A and not shown). This result indicates that in the *ipaB* and *ipaD* backgrounds, constitutive secretion of translocators is independent of MxiC.

### The needle still plays a role in signal transduction during constitutive secretion

**Fig. 4:**
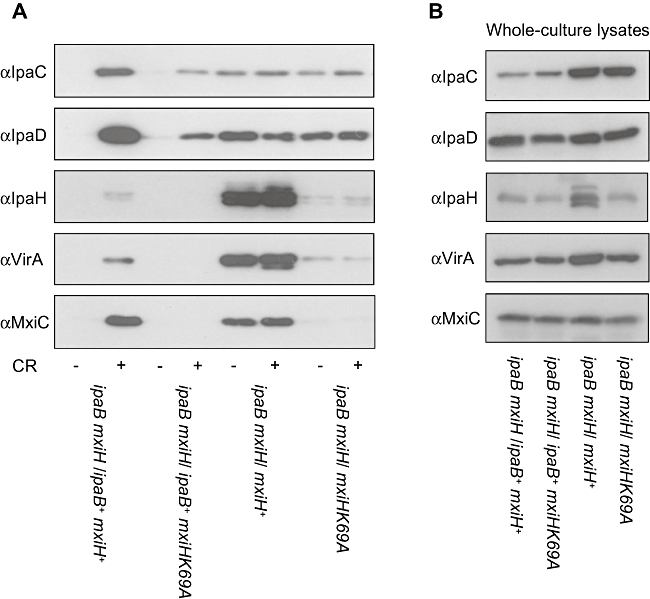
The needle is involved in MxiC release even in the absence of a functional tip complex. A. Analysis of proteins secreted after CR induction by *Shigella ipaB mxiH* double mutant complemented with the indicated plasmids. Samples were collected as in Fig. 1B and Western blotted with the indicated antibodies. B. Total protein expression levels. Samples were collected as in Fig. 2C and Western blotted with antibodies against translocator proteins IpaC and IpaD, late effector IpaH, ‘middle’ effector VirA and MxiC.

As *ipaB* and *ipaD* mutants are both constitutive secretors and have no functional tip complex (Veenendaal *et al*., 2007), we asked whether, during constitutive secretion, the needle still plays a role in signal transduction. We made an *mxiH ipaB* double mutant, complemented it with *mxiH* or *mxiHK69A* and analysed the secretion phenotypes of these strains following CR induction. All strains analysed showed similar intracellular levels of MxiC (Fig. 4B). Presence of mutation *mxiHK69A* prevents MxiC secretion in both *ipaB*^+^ and *ipaB* mutant backgrounds (Fig. 4A). Furthermore, secretion of the late effector IpaH and ‘middle’ effector VirA was drastically reduced when *mxiHK69A* was present (especially evident in the *ipaB* mutant background; Fig. 4A, third and forth panel from top). However, secretion of translocator proteins IpaC and IpaD was not significantly altered in the *ipaB mxiHK69A* background (Fig. 4A, top two panels). Altogether these results suggest that even in the presence of a profoundly altered tip complex, the needle still plays a role in MxiC release and hence in regulating effector secretion.

### T3SS assembly and needle tip composition are not altered in the *mxiC* mutant

**Fig. 5:**
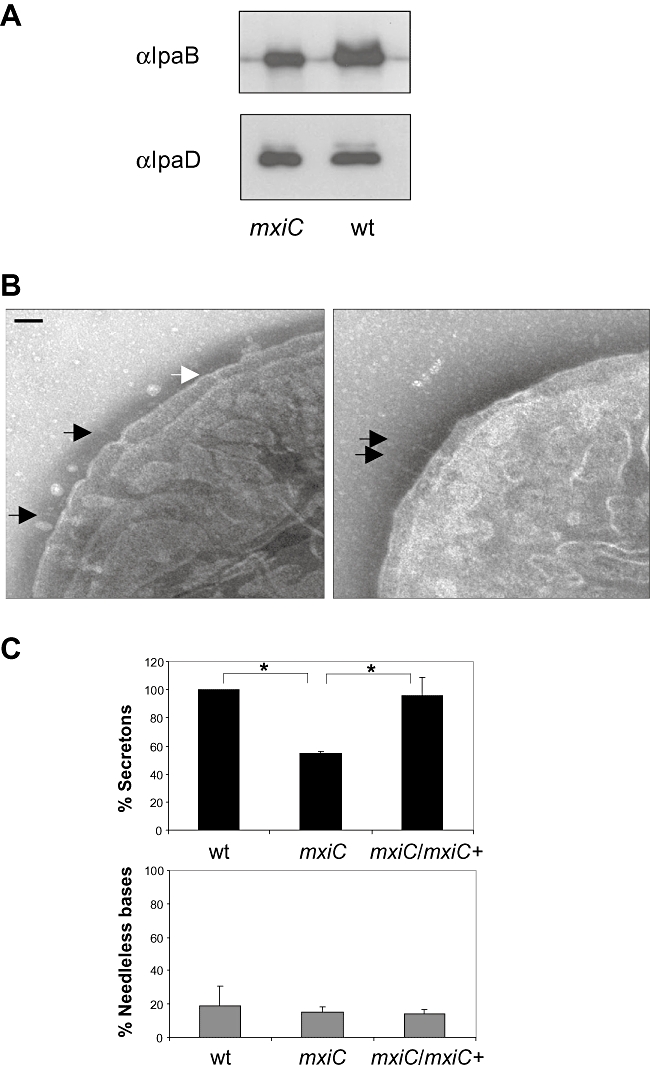
The Ipa composition of needles and the morphology of secretons are not altered in an *mxiC* mutant. A. Needles from wild-type and *mxiC* mutant strains were isolated as described in *Experimental procedures* and Western blotted with the indicated antibodies (samples were normalized for the amount of MxiH present in preparations as detected by silver-stain, not shown). B. Pictures show secretons (dark arrows) and bases (needleless secretons, white arrow) visualized by transmission EM (as described in *Experimental procedures*) for the wild-type (left) and the *mxiC* mutant (right) strains. Scale bar equals 50 nm. C. Analysis of secreton abundance and assembly. Number of complete secretons and bases quantified from electron micrographs of osmotically shocked ‘ghost’ cells prepared from the indicated *Shigella* strains. Percentages of secretons (top panel) are relative to the wild-type strain (that showed 1–2 secretons per image and is set to 100%). Percentages of bases/secretons (bottom panel) are calculated for each sample. Data are derived from two independent experiments where 20–30 images per sample where taken. Errors given are standard deviations. The asterisk ‘*’ indicates that differences with respect to the number of secretons were statistically different between the *mxiC* mutant strain and both the wild-type and complemented strains, as calculated with a Student's *t*-test (*P* < 0.05).

As secretion was altered in an *mxiC* mutant, we decided to investigate if it has a wild-type needle tip complex. To address this, we analysed the composition of needles purified from the mutant. Long needles were purified as described previously (Veenendaal *et al*., 2007) from the wild-type strain and the *mxiC* mutant overexpressing the needle protein MxiH and their Ipa content was analysed by Western blot (Fig. 5A). We found that when compared with the wild-type strain, the *mxiC* mutant has similar amounts of IpaB, IpaD and IpaC (the latter was low and is hence not shown) proteins present in needles.

In order to examine if the *mxiC* mutant could have a defect in T3SS assembly or number, cultures from the wild-type, the *mxiC* mutant and the *mxiC* complemented strains were osmotically shocked to produce ‘ghost’ cells where secretons are observable at the cell periphery (Blocker *et al*., 2001). The number of secretons and bases (secretons without needles, which can be attributed to assembly of immature and thus incomplete secretons or caused by the mechanical force applied during the preparation of ghost cells) was estimated by observation of negatively stained ghost cells using transmission electron microscopy (Fig. 5B). Although the total number of secretons in the *mxiC* mutant was decreased by nearly twofold when compared with the wild-type or complemented strains (Fig. 5C, top panel), the percentage of secretons without needle (bases/secretons) was similar for the three strains (Fig. 5C, bottom panel). Overall, these data suggest that the mutant shows no defect in T3SS assembly, although MxiC may also be involved in a mechanism that regulates the number of T3SSs in each cell.

### MxiC localizes to the cytosolic fraction

**Fig. 6:**
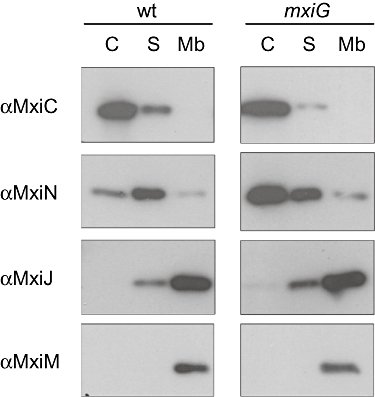
Distribution of MxiC within cellular fractions. Bacteria were fractionated as described in *Experimental procedures*. Samples were separated by SDS-PAGE and Western blotted with the indicated antibodies. Fractions are noted as C: cytosol; S: sucrose cushion and Mb: membrane. MxiN, a cytoplasmic protein that associates with the T3SS; MxiJ, a T3SS inner membrane protein and MxiM, a T3SS outer membrane protein were used as controls.

To investigate MxiC's localization, bacterial fractions from wild-type and *mxiG* strains were prepared by mechanically lysing bacteria. The clarified whole cell extract was ultracentrifuged on top of a sucrose cushion to generate three fractions: a top cytosolic fraction, a middle sucrose cushion fraction – where we expected large soluble protein complexes to migrate – and a membrane pellet. The presence of several T3SS proteins in the different fractions was analysed by Western blot. As controls, we used inner and outer membrane T3SS proteins, respectively, MxiJ and MxiM. These were detected mostly in the membrane fraction (Fig. 6), indicating that the overall fractionation procedure was successful. MxiN is a cytosolic protein that is in a large complex with the ATPase Spa47 (Jouihri *et al*., 2003; Johnson and Blocker, 2008) and also a probable component of the T3SS C-ring (Morita-Ishihara *et al*., 2006). MxiN localized to the cytosolic, sucrose cushion and membrane fractions. The proportion of MxiN in the different fractions was altered in the *mxiG* mutant strain relative to wild-type, suggesting that MxiN association with the T3SS is partially dependent on the presence of MxiG, with which it would be expected to associate at the cytoplasmic face of the inner membrane. However, MxiC was primarily detectable in the cytoplasmic fraction and undetectable in the membrane fraction. Furthermore, its distribution did not change in the absence of MxiG. This indicates that under our experimental conditions, MxiC has a largely cytosolic localization.

## Discussion

Type III secretion is a tightly regulated process initiated only after host cell contact. How the different parts of the T3SS work together to prevent secretion prior to cell contact and how the activation signal is transmitted to the cytoplasm to trigger secretion is not understood. Overall, our data support a model with gating mechanisms located at the cytoplasmic face of the T3SS apparatus that prevent secretion in the absence of activation signals. In addition, they indicate that the needle plays a key role in receiving activation signals and transmitting them to the T3SS base and the gate-keeper protein MxiC to activate translocator and then effector secretion.

YopN/TyeA from *Yersinia*, InvE and SsaL from *Salmonella* and SepL from EPEC/EHEC amongst others belong to the MxiC family of T3SS secretion regulators (Pallen *et al*., 2005). All these proteins prevent effector secretion; however, their role in translocators secretion is not clear. In *invE*, *ssaL* and *sepL* mutants, secretion of translocator is severely impaired (Kubori and Galan, 2002; Coombes *et al*., 2004; O'Connell *et al*., 2004; Deng *et al*., 2005). However, it is increased in *yopN* or *tyeA* mutants (Forsberg *et al*., 1991; Iriarte *et al*., 1998; Day *et al*., 2003; Ferracci *et al*., 2005). Recently, it was reported that secretion of translocators was not affected in an *mxiC* mutant (Botteaux *et al*., 2009). Yet, our new data suggest that in *Shigella*, MxiC does play a role in induction of translocator secretion. The *mxiC* mutant shows weak and delayed secretion of translocators in response to CR when compared with the wild-type strain (Fig. 1).

Deletion of *mxiC* in the *mxiHK69A* and *ipaBΔ3* backgrounds, which also lead to decreased translocator release, confirmed our original finding (Figs 2 and 3). We found that although the *mxiC* mutant does not show a defect in T3SS assembly, the number of secretons was decreased by 40% when compared with the wild-type strain. Therefore, it is tempting to speculate that MxiC could have a feedback role in transcriptional regulation of T3S machinery-encoding genes, as has been described for other T3SS regulators (Du *et al*., 2009). However, the decrease in the number of secretons cannot account for the defect in translocator secretion as some double mutants show this defect in secretion but not others (compare *mxiC ipaB* versus *mxiC ipaBΔ3* in Fig. 3, where both strains express similar levels of translocator proteins; however, the secretion pattern is different). Although translocator secretion was impaired in the *mxiC* mutant, it still showed a weak response to Congo red. This suggests that in *Shigella*, more than one mechanism is involved in activation of translocator secretion, with one/some still working in the *mxiC* mutant.

The *mxiC* mutant shows at 10-fold reduction in invasion efficiency compared with the wild-type strain (data not shown), as previously described (Botteaux *et al*., 2009). Botteaux and co-workers attributed that to the premature secretion by the mutant of some effectors. However, we and others have previously shown that constitutive secretion does not always correlate with a lack of invasion (Picking *et al*., 2005; Roehrich *et al*., 2010). Additionally, the *mxiC* mutant shows 60% haemolysis compared with the wild-type strain (Fig. S2). Since it has been reported that mutants lacking early effector proteins are normally haemolytic (Blocker *et al*., 1999), haemolysis depends on translocator function only. So the defect in haemolysis showed by the *mxiC* mutant is most probably due to its low level of inducible translocator secretion. We therefore suggest that the *mxiC* mutant's decreased ability to invade cells is rather due to impairment in translocator secretion and hence translocon formation. Of course, this defect could also be enhanced by the decrease in the number of secretons displayed by the *mxiC* mutant.

**Fig. 7:**
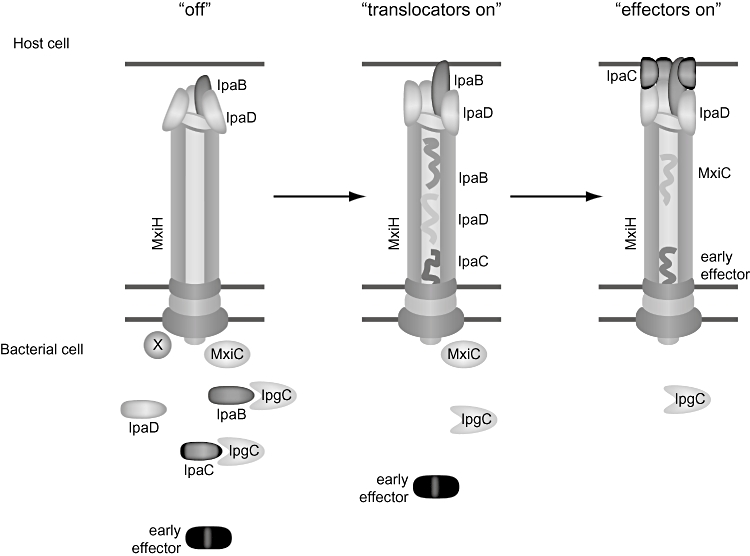
Working model for T3SS regulation of secretion in *Shigella*. Based on our results we suggest that after the T3SS completion there are three possible secretion states: ‘secretion off’, ‘translocator secretion on’ and ‘effector secretion on’. ‘*Secretion off*’ state: following assembly of the needle and its tip, our data suggest that a putative repressor mechanism (X) is put in place to prevent translocator secretion. MxiC prevents effectors secretion. ‘*Translocators on*’ state: Sensing of the host cell by the tip complex transmit a signal to the cytoplasm and translocator proteins are secreted, our data suggest that MxiC is positively involved in this step. ‘*Effector on*’ state: Our data indicate that a second signal, probably transmitted through the needle induces MxiC release and as consequence early effectors are secreted. For simplicity late effector genes and proteins are not included in the figure.

As *ipaB* and *ipaD* mutants constitutively secrete both translocator and effector proteins and neither of them have a complete tip complex, it was proposed that the needle is in a closed conformation prior to reception of the activation signal and that absence of the needle tip completely deregulates secretion (Parsot *et al*., 1995; Veenendaal *et al*., 2007). However, the *mxiC* mutant secretes effectors constitutively and yet the composition of the needle tip does not seem altered in this mutant. Evidently, we cannot exclude that needle tips from *mxiC* are in a partially/dynamically open conformation, but with unchanged composition. Still, we have shown that even in the absence of a tip complex, MxiHK69A blocks MxiC secretion and as a consequence effector secretion (Fig. 4). This strongly suggests that prevention of effector secretion prior to activation is not due to a closed conformation of the needle tip but to the presence of MxiC inside the bacteria. Therefore, taken together, our new data suggest that after completion of the needle and its tip, the positioning of the tip complex allosterically changes the conformation of the T3SS needle/apparatus and MxiC is rightly positioned to prevent effector export (Fig. 7‘secretion off’ state).

We have shown that both *ipaD* and *ipaB* mutants still constitutively secrete translocators in the absence of *mxiC*. However, we found that MxiC's function in translocator secretion is dominant in the *ipaBΔ3* mutant as when *mxiC* was deleted in this background, translocator secretion went from being constitutive to very weakly inducible, like in the *mxiC* mutant (Fig. 3). Therefore, the constitutive secretion of translocators seen in the *ipaB* or *ipaBΔ3* mutants has a different cause as only the second is MxiC-dependent. We hence hypothesize that in non-activated conditions, a mechanism that represses translocator secretion, directly or indirectly involving IpaD and/or IpaB, is put in place. After reception of the activation signal, such a repression mechanism would be counteracted and the translocators secreted. Our results strongly suggest that MxiC is involved in counteracting this repression (Fig. 7, ‘translocators on’ state). Alternatively or additionally, MxiC could play a role in optimizing the targeting of the translocators to the export apparatus. As in an *ipaD* or *ipaB* mutant the repression mechanism would not be in place, deletion of *mxiC* has no effect. In contrast, mutant *ipaBΔ3*, which is constitutively in an ‘on’ conformation (Roehrich *et al*., 2010) needs MxiC to counteract the repression mechanism. Since MxiC secretion is not needed for translocator secretion (as an *mxiHK69A* mutant that is not able to secrete MxiC has no defect in translocator secretion), MxiC must be acting from inside the cell at this point. Therefore, we presume that the repression mechanism is also intracellular. LcrV, the *Yersinia* IpaD homologue, regulates secretion through interaction with the negative regulator protein LcrG, its intracytoplasmic chaperone. However, unlike IpaD, LcrV acts as a positive regulator, as an *lcrV* mutant shows a defect in effector secretion (Nilles *et al*., 1997; Matson and Nilles, 2001). Yet, very recently it was shown that the *Pseudomonas* LcrV homologue PcrV acts as a negative regulator of effector secretion (Lee *et al*., 2010). These authors propose that PcrV controls effector secretion by assembling into a functional needle tip complex. Interestingly, they also found that the LcrG homologue, PcrG, acts independently of PcrV and from within the cytoplasm to prevent premature effector secretion. Our PcrV/LcrV homologue IpaD carries its own chaperone at its N-terminus (Johnson *et al*., 2007). IpaD deletion mutants within this region still bind the needle tip yet demonstrate constitutive secretion (Picking *et al*., 2005; Espina *et al*., 2006; Veenendaal *et al*., 2007).

The isolation of needle mutants showing an altered tip complex and secretion phenotype led us and others to propose that the needle and its tip are involved in regulation of T3SS activity (Kenjale *et al*., 2005; Torruellas *et al*., 2005; Davis and Mecsas, 2007; Veenendaal *et al*., 2007). Here we have moved a step forward in our molecular understanding of how the activation signal is transduced to the cytoplasm in finding that the needle plays a role controlling MxiC release after translocator secretion has occurred. We have demonstrated that the inability of the needle mutant *mxiHK69A* to secrete effectors is due to its defect in MxiC secretion and that even in an *ipaB* mutant background (otherwise a constitutive secretor) presence of MxiHK69A prevents MxiC secretion and as a consequence effector secretion (Fig. 4). Therefore, the simplest hypothesis is that after translocator secretion, a second activation signal is transmitted through the needle to release MxiC (Fig. 7‘effectors on’ state). This signal allows effector secretion and cannot be transmitted through MxiHK69A needles. Alternatively or additionally, the presence of MxiHK69A could provoke a conformational change in the base of needle complex that could strengthen a putative interaction of MxiC with the T3SS, preventing its secretion. Finally, after MxiC release from the T3SS, early effectors are secreted. As previously reported, secretion of the early effector OspD1, which acts as a repressor of the transcriptional activator MxiE, together with the increase in the concentration of free IpgC (the chaperone of secreted translocators IpaB and IpaC), will then activate the expression of late effector genes (Parsot *et al*., 2005; Botteaux *et al*., 2009).

In our view, the evidence for two activation signals is now strong and its existence illustrates how exquisitely sophisticated T3SSs are. The nature of both activation signals remains unknown but logically the first one should indicate host cell membrane contact (Veenendaal *et al*., 2007) and the second should somehow indicate that a functional translocon has been assembled at this location in order to allow secretion of the effectors directly into the host cell. Recently, it has been shown that sensing of cytoplasmic pH by intracellular *Salmonella* causes degradation of the SsaM/SpiC/SsaL regulatory complex, where SsaL is an MxiC homologue, inducing secretion of effector proteins (Yu *et al*., 2010). These authors found that a translocon deletion mutant responds to the pH shift, indicating that the sensor is not part of the translocon and suggesting that could instead be the needle subunit itself or a protein localized towards the base of the secretion apparatus (Yu *et al*., 2010).

How MxiC and homologues prevent effector secretion remains unclear in all systems. Botteaux and co-workers reported an association of MxiC with Spa47 (Botteaux *et al*., 2009), therefore MxiC's interaction with the T3SS may occlude an acceptor site for effector proteins. MxiC fractionates overwhelmingly into the cytosolic fraction (Fig. 6). In addition, there is some controversy about the localization of SepL, the EPEC and EHEC MxiC homologue: in EPEC SepL was found to be cytosolic (O'Connell *et al*., 2004) or in both the cytoplasm and the inner membrane (Deng *et al*., 2005), in EHEC it was detected in both cytosolic and membrane containing fractions (Kresse *et al*., 2000). Therefore, interactions between MxiC and other proteins within the T3SS apparatus may be transient and hence lost during the fractionation procedure. Alternatively, at steady state perhaps most MxiC is indeed cytosolic but the few molecules that interact with T3SSs account for its function under non-activated conditions. In fact, SsaL, the *Salmonella* SPI-2 MxiC homologue, was recently found to form part of a regulatory complex that dissociates from the membrane in response to an increase in pH leading to effector secretion (Yu *et al*., 2010). Although SsaL localizes mostly to the cytoplasmic fraction, a small proportion of the membrane bound protein co-precipitates with the membrane associated complex and importantly, it is this fraction that dissociates from the membrane upon pH increase (Yu *et al*., 2010).

As a prerequisite to understanding how MxiC regulates T3SS activation, a detailed examination of its molecular interaction partners within mutants blocked at different stages in the secretion activation process will have to be performed. Our aim for the future will be to use the available atomic structure of MxiC (Deane *et al*., 2008) to understand precisely how MxiC acts to regulate T3SS secretion.

## Experimental procedures

### Bacterial strains, plasmids and primers

Table 1 lists the strains and plasmids used in this study. *Shigella flexneri* strains were grown in trypticase soy broth (Becton Dickinson) at 37°C with the appropriate antibiotics at the following final concentrations: ampicillin 100 µg ml^−1^, kanamycin 50 µg ml^−1^, tetracycline 15 µg ml^−1^.

**Table 1:** Bacterial strains and plasmids used in this study.

*Shigella* strains	Strain/plasmid	Reference
M90T	Wild-type M90T, serotype 5a	Sansonetti *et al*. (1982)
SF620	*ipaB* mutant	Menard *et al*. (1993)
*mxiG*	*mxiG mutant*	Allaoui *et al*. (1995)
*mxiC*	*mxiC* KO mutant	This study
*mxiC/mxiC*^+^	*mxiC* mutant/ pIMA208	This study
SH116	*mxiH* mutant	Blocker *et al*. (2001)
*mxiHK69A*	SH116/pRKmxiHK69A	Kenjale *et al*. (2005)
*mxiHD73A*	SH116/pRKmxiHD73A	Kenjale *et al*. (2005)
*mxiC mxiH*	Double mutant *mxiC mxiH*	This study
*mxiC mxiHK69A*	*mxiC mxiH*/pRKmxiHK69A	This study
*ipaBΔ3*	SF620/pDR2	Roehrich *et al*. (2010)
*mxiC ipaB*	Double mutant *mxiC ipaB*	This study
*mxiC ipaBΔ3*	*mxiC ipaB*/pDR2	This study
*ipaB mxiH*	Double mutant *ipaB mxiH*	This study
*ipaB mxiH/ipaB*^+^*mxiH*^+^	*ipaB mxiH*/pIMA216	This study
*ipaB mxiH /ipaB*^+^*mxiHK69A*	*ipaB mxiH*/pIMA218	This study
*ipaB mxiH /mxiH*^+^	*ipaB mxiH*/pRKmxiH	This study
*ipaB mxiH /mxiHK69A*	*ipaB mxiH*/pRKmxiHK69A	This study
M90T/ pRK2*mxiH*	Wild-type /pRK2*mxiH*	Veenendaal *et al*. (2007)
*mxiC/*pRK2*mxiH*	*mxiC*/pRK2*mxiH*	This study

Plasmids	Description	Reference
pDR1	pUC19*-ipaB*	Roehrich *et al*. (2010)
pDR2	pUC19*-ipaB* C-terminal three-amino-acid truncation	Roehrich *et al*. (2010)
pIMA208	*mxiC* gene cloned into pUC19	This study
pIMA216	*ipaB* and *mxiH* genes cloned into pUC19	This study
pIMA218	*ipaB* and *mxiHK69A* genes cloned into pUC19	This study
pKD46	Plasmid expressing lambda red recombinase	Datsenko and Wanner (2000)
pRK*mxiH*	*mxiH* cloned into a pUC18 derivative	Kenjale *et al*. (2005)
pRK*mxiHK69A*	*mxiHK69A* cloned into a pUC18 derivative	Kenjale *et al*. (2005)
pRK*mxiHD73A*	*mxiHD73A* cloned into a pUC18 derivative	Kenjale *et al*. (2005)
pRK2*mxiH*	Plasmid overexpressing mxiH	Kenjale *et al*. (2005)

### Construction of non-polar knockout mutant strains

The *mxiC* mutant was generated by using the Lambda Red system as described by Datsenko and Wanner (2000). Briefly, to replace the wild-type *mxiC* gene, a tetracycline resistance cassette with 50 pb flanking regions homologous to the *mxiC* gene was amplified from strain TH2788 (Frye *et al*., 2006) using the primers mxiC_KO_tetF and mxiC_KO_tetR (Table 2). *Shigella flexneri*, serotype 5a strain M90T carrying plasmid pKD46 (encoding the Red recombinase genes), was grown at 30°C to an OD_600_ of 0.2 and then induced by adding arabinose to a final concentration of 0.2% during approximately 2 h. When cells reached an OD_600_ of ∼ 0.6, they were made electrocompetent by concentrating them 100 times and washing them 3 times with ice-cold water. They were electroporated with 1 µg of the purified PCR product described above. Mutants where the *mxiC* gene has been replaced by the tetracycline cassette were selected on plates with tetracycline. The same procedure was used to inactivate *mxiC* in strains SF620 (*ipaB*) and SH116 (*mxiH*) giving rise to strains *mxiC ipaB* and *mxiC mxiH* respectively. To construct strain *ipaB mxiH*, the *ipaB* gene was exchanged for a tetracycline cassette in the single mutant strain SH116 (*mxiH*) using the same method. All gene replacements were confirmed by sequencing.

**Table 2:** Primers used in this study.

Primer	Sequence^a^
mxiC_SalI	GCACGCGTCGACAACTATAAAGTAGGTGA TGTATGCTTG
mxiC_BamHI	CGCGGATCCCTGGATCACTTTTATCTCCTGTTATC
mxiH_RBS_PstI	ACTACTGCAGCTAACAGGAGGAATTACATATGAGTGTTACAGTACCGAATGATG
mxiH_XbaI	ACTATCTAGATTATCTGAAGTTTTGAATAATTG
mxiC_KO_tetF	CATTGGTTTCATACTTAAATTACTAACTATAAAGTAGGTGATGTATGCTTGATGTTAATTAAGACCCACTTTCATAATTAAGACCCACTTTCA
mxiC_KO_tetR	TGCTTAAGAAAAGACTGGATCACTTTTATCTCCTGTTATCTAGAAAGCTCCATATGAATATCCTCCTTACTAAGCACTTGTCTCCTG

a Restriction sites are underlined.

### Construction of plasmids

To complement the *mxiC* mutant, *mxiC* was amplified by PCR from the M90T virulence plasmid pWR100 using primers mxiC_SalI and mxiC_BHI (Table 2) and cloned into pUC19 digested with SalI and BamHI giving rise to plasmid pIMA208. pIMA216 and pIMA218 encoding *ipaB* and *mxiH* or *mxiHK69A* are derivatives of pDR1 that contains *ipaB* cloned into pUC19 (Roehrich *et al*., 2010). *mxiH* or *mxiHK69A* were amplified by PCR from pRK*mxiH* or pRK*mxiHK69A* (Cordes *et al*., 2005; Kenjale *et al*., 2005) using primers mxiH_RBS_PstI and mxiH_XbaI. The digested PCR fragments were cloned into pDR1 digested with PstI and XbaI (downstream of the *ipaB* gene). All plasmids were verified by sequencing.

### Analysis of protein synthesis and secretion

#### Total level of protein expression

*Shigella flexneri* strains were grown at 37°C until exponential growth (OD_600_ ≈ 1) was reached. Samples of the cultures, representing the total protein fraction, were denatured in Laemmli sample buffer, separated by SDS-PAGE and used for Western blot analysis.

#### Overnight leakage

*Shigella flexneri* strains were grown overnight at 37°C. Bacteria were collected by centrifugation at 15 000 *g* for 10 min at 4°C. The ‘stationary phase’ supernatants were denatured in Laemmli sample buffer, subjected to SDS gel electrophoresis and Silver-stained (Silver Xpress kit, Invitrogen).

#### Congo red induction

Bacteria collected during exponential growth (OD_600_ ≈ 1) were resuspended at OD_600_ = 5 in phosphate-buffered saline. Congo red was added (final concentration of 200 µg ml^−1^) to induce T3SS activity. After incubation at 37°C for 15 min, the samples were centrifuged at 15 000 *g* for 10 min at 4°C and the supernatants were denatured, separated by SDS-PAGE and Silver-stained or used for Western blot analysis.

Mouse monoclonal antibodies (Mabs) used for Ipa protein detection have been described previously and were generous gifts from Armelle Phalipon (αIpaB, known as H16, and αIpaC, a mixture of two Mabs known as K24 and J22) and Kirsten Niebuhr (αIpgD) (Blocker *et al*., 1999). The rabbit polyclonal sera against IpaH (Mavris *et al*., 2002) and IpaD (Blocker *et al*., 1999) are also published and were kind gifts of Regis Tournebize and Claude Parsot respectively. The MxiC rabbit polyclonal antibody was raised (Eurogentec, Belgium) against a fragment of MxiC, purified as a recombinant polypeptide encompassing MxiC residues 74–355 and carrying an N-terminal His tag with a thrombin cleavage site (Deane *et al*., 2008), which was a kind gift from Janet Deane and Susan Lea. The rabbit polyclonal anti-MxiN serum is described in Johnson and Blocker (2008), the mouse monoclonal antibody against MxiJ and the affinity purified polyclonal anti-MxiM rabbit antibodies in Zenk *et al*. (2007).

### Electron microscopy

Ghost cells were prepared by osmotic shock treatment of bacterial cultures grown to an OD_600_ of 1 using glass beads as described previously (Kenjale *et al*., 2005). Samples were deposited onto 300-mesh, Formvar and carbon-coated glow-discharged copper grids and subsequently stained for 1 min with 2% phosphotungstic acid at pH 7. Electron microscopy images were taken on a Tecnai 12 transmission electron microscope (FEI) operating at 120 KeV. Micrographs were recorded at a magnification of 20 000 on an Eagle 4K × 4K CCD camera (FEI) using the TIA software (FEI). Secretons and bases were counted by hand.

### Needle preparations

Needles were purified from strains containing the pRK2*mxiH* plasmid as previously described (Cordes *et al*., 2005), in the absence of T3SS activation. Isolated needles were passed over a Superdex S200 HR16/30 column equilibrated in 20 mm Tris-HCl pH 7.4, 150 mm NaCl. One-millilitre fractions were collected and precipitated with trichloroacetic acid (TCA). Samples were separated by SDS-PAGE and Silver-stained to normalize the amount of MxiH prior to Western blotting with appropriate antibodies.

### Fractionation of bacteria

Around 400 ml of bacteria was grown to an OD_600_ of 1 and pelleted by centrifugation at 4000 *g* for 10 min. Pellets were resuspended in 10 ml of Tris buffer (20 mm Tris, 150 mm NaCl, pH 7.4) and cells were lysed by two passages through a French Press at 10 000 Pa and a few crystals of DNase I were added to reduce sample viscosity. Unbroken cells were removed by several rounds of centrifugation (10 min at 10 000 *g*), until no further pellet was obtained. Cell lysates (0.66 ml) were added to a TLS 55 Ultraclear centrifuge tube (Beckman, 347 356) containing 20% w/w sucrose solution (1.54 ml). Membranes were pelleted by ultracentrifugation at 200 000 *g* for 1 h at 4°C in an Optima TLX Ultracentrifuge (Beckman). Three fractions were collected, the first two by always rotating the collection pipette slowly around the tube's edge at the solution's meniscus: the top, cytosol fraction (0.6 ml, the last 0.06 ml being discarded), the middle, sucrose cushion fraction and the bottom, membrane pellet, which was first carefully washed with water and then resuspended in 0.6 ml Tris buffer. The sucrose fraction was precipitated with 10% trichloroacetic acid according to conventional procedures and finally resuspended in 0.6 ml Tris buffer. Five microlitres of each of the samples was separated by SDS-PAGE and used for Western blot analysis.
