# Supplementary material for: The Shigella T3SS needle transmits a signal for MxiC release, which controls secretion of effectors
Source: Mol Microbiol. 2010 Oct 11;78(6):1365–78. doi: 10.1111/j.1365-2958.2010.07413.x (PMC3020320; doi:10.1111/j.1365-2958.2010.07413.x)
Supplement: Supplementary file 1 [file mmi0078-1365-SD1.pdf]

Supporting material for

The *Shigella* T3SS needle transmits a signal for MxiC release, which controls secretion of effectors

Isabel Martinez-Argudo*, A. Dorothea Roehrich and Ariel J. Blocker

Schools of Cellular and Molecular Medicine and Biochemistry, University of Bristol, BS8 1TD, United Kingdom

*Corresponding author: School of Cellular & Molecular Medicine

University of Bristol. University Walk, BS8 1TD, United Kingdom Tel: +44-1173-312-059;

Fax: +44-1173-312-091; E-mail: I.Martinez-Argudo@bristol.ac.uk

Supporting Experimental procedures

Constructions of plasmid and strains

Plasmid pIMA227 was constructed by cloning a 1 kb *Sal*I-*Kpn*I fragment containing the entire *mxiC* gene (including its ribosome binding site) into the IPTG inducible vector pACT3 (Dykxhoorn *et al.*, 1996). pIMA227 was transformed into the *mxiC* mutant (strain *mxiC/pACT3mxiC*) and MxiC expression was titrated using IPTG.

Contact hemolysis

Contact hemolysis was performed as previously described (Blocker et al., 1999). Briefly, 100 μl of freshly prepared red blood cells (5×10^{8} ml^{-1}) were mixed with 100 μl of exponentially grown bacteria at an MOI of 20 in a 96-well rounded plate, centrifuged and incubated at 37°C 1h. The cells were then resuspended, centrifuged and 100μl of the supernatant transferred to a new plate were the optical density at 405 nm was measured. The percentage of hemolysis (P) was calculated using the equation P: [(X-B)/ (T-B)] x100, where X is the optical density value of the sample analysed; B is the baseline of the assay and was set with red blood cells (RBCs) incubated with Tris-saline instead of bacteria; T is total hemolysis and was the value obtained when RBCs were incubated with Tris-saline containing 1% Triton X-100.

Figure S1

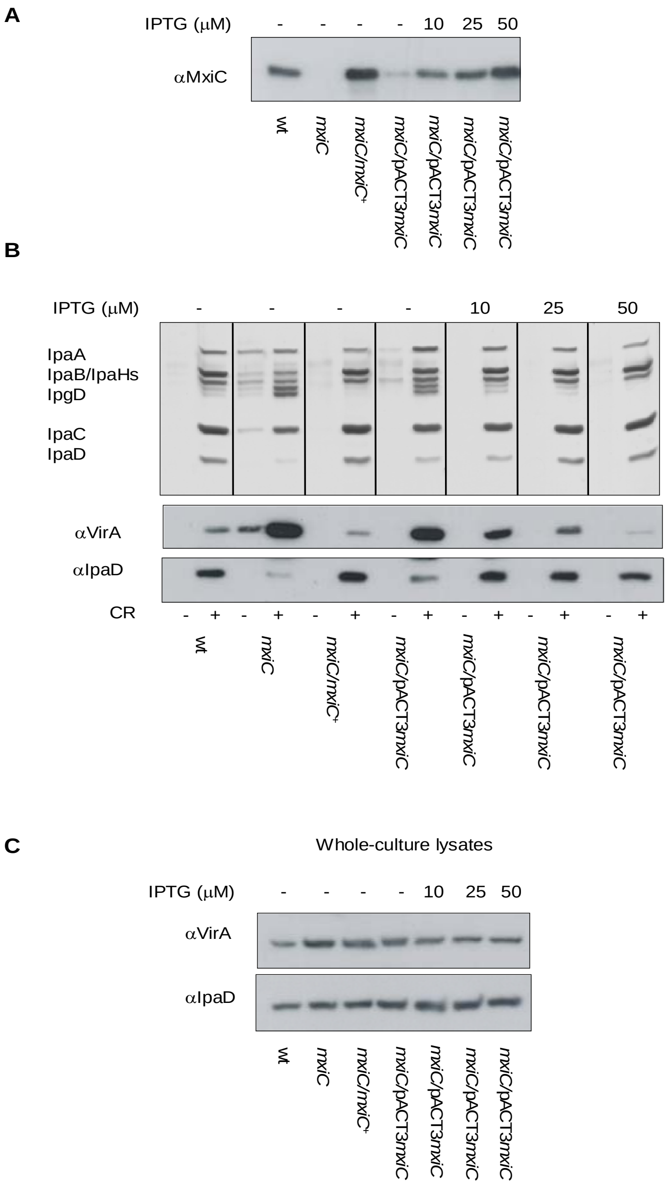

Fig. S1. Complementation of the *mxiC* mutant with an IPTG inducible plasmid (A) Total protein expression level from *Shigella* wild-type (wt), *mxiC* mutant and complemented strains (*mxiC/mxiC*$^{+}$: *mxiC* mutant complemented with *pUCmxiC*; *mxiC/pACT3mxiC*: *mxiC* mutant

complemented with *pACT3mxiC*). Samples were taken from exponentially grown cultures (growing in the absence or indicated IPTG concentrations), normalised according to cell density, separated by SDS-PAGE and Western-blotted with an antibody against MxiC. (B) Protein secretion in response to Congo red induction (CR). Cultures were grown to exponential phase, normalised by optical density and supernatants were collected from duplicates incubated 15 min at 37°C with or without the artificial inducer CR, separated by SDS-PAGE and silver-stained (top panel) or Western-blotted with antibodies against effector VirA and translocator IpaD (bottom panels). (C) Total protein expression level. Samples were collected as in (A) and Western-blotted with the indicated antibodies.

Figure S2

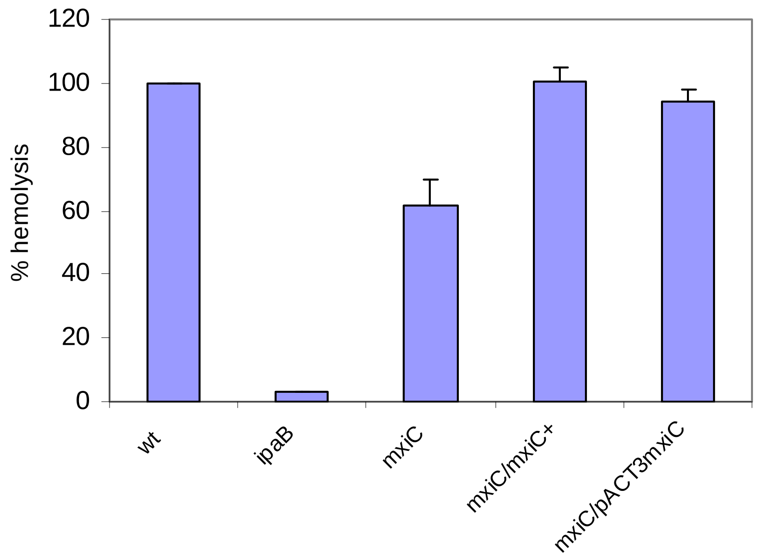

Fig. S2. The *mxiC* mutant shows a defect in contact hemolysis. Hemolysis is shown as percentage of the wild-type strain that showed 88 % hemolysis and is set to 100%. *mxiC/mxiC*$^{+}$: *mxiC* mutant complemented with *pUCmxiC*; *mxiC/pACT3mxiC*: *mxiC* mutant complemented with *pACT3mxiC* and grown in the presence of 25µM IPTG.

The values are averages of two independent experiments performed in triplicate. Errors given are standard deviations. Differences with respect to the percentage of hemolysis were statistically different between the *mxiC* mutant and all the other strains as calculated with a Student t-test ($p < 0.05$).

References

Dykxhoorn, D.M., St Pierre, R., and Linn, T. (1996) A set of compatible tac promoter expression vectors. *Gene* **177**: 133-136.
